# Diseased Food and Disease
*Report on Murrain in Horned Cattle; the Public Sale of Diseased Animals; and the Effects of the Consumption of their Flesh on Human Health. By E. Headlam Greenhow, M.D. Blue Book. London: 1857.Two Letters to the Right Hon. Sir George Grey, on the Cattle Plague and Diseased Meat in their Relations with Public Health and the Interests of Agriculture. By Joseph S. Gamgee. London : Richards. 1857.Reports on the Relations of Food and Disease. *Medical Times and Gazette* for 1857, Nos. 354, 355, 356, 357, 358, 360. London: Churchill.Letter on the Apprehended Murrain. Ry John N. Radcliffe. *Leeds Mercury*, April 28th, 1857.Various Letters in the *Times* newspaper on Murrain. 1857.


**Published:** 1857-07

**Authors:** 


### DISEASED FOOD AND DISEASE.*

In the first number of this Review we introduced to the notice of the public the results which had been arrived at by M. Soumille, of Avignon, regarding the characters and effects of deceased animal food. At that time but little was heard in England on these important questions, but within the last four months there has been an entire revolution, and now the subject is literally and practically in everybody's mouth. A variety of circumstances have led to this change of feeling. The first noteworthy facts consisted in the promulgation of the modern views as to the origin of diseases of the entozoic class, from the consumption of improper animal food. Then came a report on diseased food, by the Association of Medical Officers of Health. Then came news of a cattle murrain on the continent—then rumour of a cattle murrain here—then vague notions of human plagues, of a great or insidious kind, springing out of dinners eaten in the bliss of ignorance. These rumours, and rumours of rumours, are ever the forerunners of the seasons which string up communities to the listenings of their destinies. These are the birthdays of prophets and thunder mortals, in which, whoever enjoys the sight of a general kneeshaking, may fill himself with fun to the brim. Blow the trumpet loud and fierce :—give it double utterance, give it shake, give it extreme modulation, make it curse, make it warn, make it threaten, make it whisper, make it sigh, make it spit, give its sounds echoes, give the echoes encouragement ! and oh ! great artist, with your lips at the instrument, whether your music be the music of Solon, or of Francis Moore, physician, depend on it, you will get an audience, and an attentive ear.

When the time was ripe for the event, and a declaiming seer was publicly required to pronounce anathema on fever cows, Mr. Gamgee put on the garb of Hygienic high priest. In this imposing figure he did what no high priest has done before. He took the trumpet we have spoken of, and blew it

---

* Report on Murrain in Horned Cattle; the Public Sale of Diseased Animals; and the Effects of the Consumption of their Flesh on Human Health. By E. Headlam Greenhow, M.D. Blue Book. London: 1857.

Two Letters to the Right Hon. Sir George Grey, on the Cattle Plague and Diseased Meat in their Relations with Public Health and the Interests of Agriculture. By Joseph S. Gamgee. London: Richards. 1857.

Reports on the Relations of Food and Disease. *Medical Times and Gazette* for 1857, Nos. 354, 355, 356, 357, 358, 360. London: Churchill.

Letter on the Apprehended Murrain. By John N. Radcliffe. *Leeds Mercury*, April 28th, 1857.

Various Letters in the *Times* newspaper on Murrain. 1857.

so terrifically, that, from 37 Great Queen Street, its noise reached the Hebrides. He made it shake, curse, warn, sigh, whisper, spit, and threaten. Never, since the days of Vespasian, and of the son of Ananus, was there such an executive or dismal scream. Woe, woe to the shambles! woe to Islington Market! woe to Mr. Nice, who winks at the nasty! woe to Mr. Pocklington! woe, woe to Newgate! woe to Mr. Fisher! woe, woe, woe to the Veterinary College! woe to fever cows! woe to the New Cattle Market! and woe to the Home Secretary himself, if he listen not to the trumpet—if he listen not to the trumpet!

Then come the echoes: the diseased meat question is suddenly exalted into the great sanitary question of the day: the cook is torn from her butcher: and butchers in general become poetical and remonstrative.

"Gin a body meet a body,
In a typhus state;
Gin a body kill a body,
Need a body eat?"

Old maidens go in for a general fast—old gentleman go out to select their own viands. Several charitable institutions put off their dinners: the lord mayor and aldermen pass weepingly over to the vegetarians: the awful conflagration tobacco clamour ends in smoke, and out of its ashes the Phœnix rises whose muscle is of beef and whose liver is of mutton.

The press expounds. The *Times* has an eruption of letters. The *Medical Times and Gazette* takes on emetic virtues. With the coolness of the giant warrior chained to the North Pole, and wanting altogether in the finer sympathies and more exquisite sentiments, this ruthless periodical writes down statements which are well calculated to turn all flesh into grass and all grass into flesh. Lastly, but not leastly, Dr. Greenhow enters on the field. His book bears the blue stamp of authority; and we have it on the statement of its very title-page, that the Queen herself commanded its presentation to the Parliament—a rather important hint, one would think, to the royal purveyors.

From information up to the present date, it does not appear that the Home Secretary has carried out any of the important steps proclaimed by Mr. Gamgee. The cattle markets remain unreformed, and still the lieges who cannot buy good meat buy bad. There is, in fact, no accounting for taste. In this position of affairs, let us review calmly the facts which have been collected, without reference to the out-door clamour or the woes.

The Vienna correspondent of the *Times* enumerates three complaints, under the head of cattle plagues. 1. A catarrhal affection. 2. A pulmonary complaint, with typhoid symptoms. 3. A highly contagious typhus, which is the real cattle plague or murrain of the steppe. Dr. Greenhow enumerates more forms of general cattle disorder, viz., the *pulmonary murrain*, or lung disease, which was preceded by the *eruptive murrain*, and which has resemblances with both hooping-cough and influenza in the human subject, but differs from both in its inflammatory character; *steppe murrain*, or *rinderpest*, decribed as a febrile disease analogous to continued fever in the human subject; *dysenteric murrain*, which approaches to the Asiatic cholera in the human subject; and *carbuncular murrain*, considered by German writers as a typhus disease originating in horned cattle, but communicable to other animals, and even to man. In the conclusion of his report, Dr. Greenhow gives the following *résumé*:—

"I. *Pulmonary Murrain.*—1. The cattle disease I was desired to investigate is not of recent origin, but has prevailed among the horned cattle of the United Kingdom for the last fifteen or sixteen years.

"2. It is not peculiar to London, but is generally prevalent in country districts, and among cattle in pastures as well as in those that are exclusively stall-fed.

"3. It is probably infectious, but is also developed spontaneously in consequence of some unknown peculiarities of breed, management, season, or locality, and is not supposed to have been imported into the United Kingdom.

"4. It is identical with the *Lungenseuche* or pulmonary murrain now or lately prevailing in Mecklenburg, Holstein, and other continental countries,

"5. It has no affinity with the disease called in Germany *Rinderpest* or steppe-murrain, with which it has been often confounded by writers in this country.

II. *Murrain on the Continent.*—1. Steppe-murrain is probably more highly contagious than pulmonary murrain, and is said only to originate spontaneously among cattle of the Podolian breed, and in the steppe countries of southern Russia.

"2. Steppe-murrain is probably at present not more than usually prevalent in the south-eastern countries of Europe, and has on former occasions often prevailed more extensively than now in Austria and Prussia.

"3. The only probable chance of the importation of steppe-murrain into the United Kingdom is by way of Prussia. It has recently shown itself at a few places only on the eastern frontier of that country, and has in each case been speedily exterminated by the active measures adopted by the authorities to secure its extinction.

"4. The regulations enforced by the authorities to exclude steppe-murrain from the Prussian territory, and to extinguish it when it is imported thither, are of so stringent a character as to render its introduction into the United Kingdom by way of Prussia unlikely.

"5. Any measures which it may be considered necessary to adopt for the exclusion of this disease from the United Kingdom must necessarily be of a permanent and not of a merely temporary character, as steppe murrain is always more or less prevalent in countries adjoining to the eastern frontiers of Austria and Prussia. Perhaps the case would be satisfactorily met if the importation of cattle were prohibited, except from countries which, as regards steppe-murrain, have clean bills of health, and require similarly clean bills from other countries importing to them.

"III. *Sale of Diseased Meat.*—1. Meat derived from animals suffering with pulmonary murrain, and probably with other diseases, is commonly and extensively sold both in London and elsewhere for human food.

"IV. *Effects on Human Health of using the Flesh of Diseased Animals.*—1. There is no satisfactory proof that the consumption of meat derived from diseased cattle, has in this country been productive of direct injurious consequences upon those who have eaten it.

"2. There are reasons for supposing that the use of meat from animals suffering from diseases unknown among the cattle of the United Kingdom has abroad frequently been attended with serious consequences on human health.

"3. The consumption of meat undergoing decomposition has frequently been injurious, and such meat cannot be eaten with safety even when cooked."

To English readers of the omnivorous class, these final deductions of Dr. Greenhow will be satisfactory. He differs in some particulars from Mr. Gamgee in relation to the number of diseased animals sold for immediate consumption in the London market, by indicating that while the number of diseased animals in the market are comparatively few, those to be met with there are not always intended at once for the shambles, but for transportation to rural districts, where they are fed and are brought into an improved condition before being sold for human consumption. We cannot but accept Dr. Greenhow's report as a valuable document, and as an improvement on works of the blue book order.

Mr. Gamgee's two letters are rather of a suggestive political, than of a scientific kind. His suggestions for the suppression of traffic in diseased animal food are as follow :—

"1. Inspectors should be on duty in the great live and dead meat markets whenever business is being carried on,—particularly at night, which, honest people should remember, is busy day with rogues;—and a multitude of rogues live in quasi-opulence by practising frauds on the food of the people; on no part of it so much as on meat.

"2. Inspectors of nuisances in the metropolitan districts should be provided in number and character proportionate to the duties to be performed. Thus, for instance, it being preposterous to suppose that the 195,000 inhabitants of St. Pancras parish can be protected from filthy and abominable frauds by the one inspector now attached to the district, sufficient provision should be made.

"3. No cattle should be allowed to land on our shores without a clean Bill of Health, which Bill the British consuls at the places of export should be instructed to furnish only upon reliable information, such as could be gleaned from the excellent veterinary schools which abound on the continent. In such matters partial security only engenders false confidence; the guarantee must be the most complete that can be given under the circumstances."

We have already commended the unflinching courage with which Mr. Gamgee carries out his views.

The articles in the *Medical Times and Gazette* are intended to show the relations which diseased food holds to disease. In a preliminary paper, a variety of basic questions are candidly put forward. Here are two examples:—

"Allowing for a moment that the flesh of diseased animals received into the human body has the effect of operating as a poison, what are the modifications of symptoms which it induces, as compared with the original symptoms in the diseased animal? What positive relations do the epidemics in the lower animals bear to the epidemics in man? What modifications in type are produced in the passage of the epidemic disorder from an animal of one class to an animal of another class? Some flickering light on this point, in reference to small-pox and the cow, and cow-pox and man, is all that relieves the darkness of science here at the present time.

"Another point. We will not dream of going back to the efficient causes of epidemics; but we would propound this first and broad inquiry. Is the propagation of epidemic disorders limited to the animal kingdom? Are all the germs of epidemics formed and circulated only in the animal domain? Or does the epidemic phenomenon take a wider root; can it be traced back to the vegetable world? Again, can an epidemic arise spontaneously, as from causes external, *i. e.* independently altogether of the idea of simple propagation of animal or vegetable transmission? Can variations of heat, of electricity, of humidity, excite any special disease which, once communicated to man or animal, shall be communicable to other men and animals?

"In the absence of labours bearing on these all-important and primary inquiries, the so-called science of epidemiology is no science at all, but a perplexing, chaotic record of confusion."

In the subsequent papers the writers of the *Medical Times and Gazette* enter more into detail on the questions before them. They produce in full Renault's experimental conclusions, which in the main go to prove that—

"Conceivable as is the repugnance felt by men at feeding upon flesh, milk, etc., which are the products of animals affected with contagious disease, there is really no danger incurred by his eating the flesh when cooked, or the milk when boiled."

They argue from other testimony and from circumstantial evidence,—

"That the flesh and viscera and milk of animals which die of *miasmatic* or *parasitic* diseases, or are killed during their progress, are unwholesome, and unfit for human food; and that when persisted in they tend to produce dysentery, diarrhœa, or scurvy amongst the people, or diseases which belong to the *dietic* class of zymotic diseases, and which require a persistent use of the pernicious food and some time for their development."

The article on milk contains the following facts, as to what are to be considered the standard properties of good milk newly drawn:—

"The fluid should be of alkaline reaction: this applies to the milk of all animals. The specific gravity should be about 1030: this applies to the milk of the woman and to that of the cow. The taste should be slightly sweet, and free from saline qualities. The colour should be of a yellowish white, opaque, free from blueness on the one hand, and marked yellowness on the other. Microscopically it should present the well known fat-globules, which average about $\frac{1}{4000}$ of an inch in diameter, and are soluble in ether, but insoluble in caustic potash. These globules should float freely over each other, and present no tendency to adhere. All granular bodies having a viscid appearance and a tendency to agglutinate should be absent, as well as globules having unequal borders and dotted surfaces, or the ordinary red corpuscles of blood. The odour of milk should be slightly aromatic, free from pungency, and from any kind of fetor or acidity."

Disordered conditions of milk may arise, continues the writer, from two sets of causes—viz., from modifications in the natural constituents of milk, or from the presence of foreign substances. Milk may thus be acid in reaction—or super-alkaline; it may contain too much water and too little casein, butter, or sugar; it may receive a special flavour from the food on which the cow is fed; it may contain an animalcule, the vibrio cyanogenus, which gives a blue colour, or the vibrio xanthogenus, which gives to it a yellow colour; it may contain blood, or pus, or those peculiar fat globules, called colostrum corpuscles, which naturally occur in milk for a few days after calving—"beastings," as it is called in common parlance.

Reviewing fairly the labours of our cotemporary, we have to thank him for many new and important truths on the nature of the diseased conditions to which food is subjected. Here the *Medical Times and Gazette* has laid a groundwork which was necessary. Further than this its writers have not yet progressed; and such few and desultory facts as have been collected on the question of diseased food in the production of disease, are not as yet sufficient to controvert M. Renault's general conclusion. The writer on milk proves, however, clearly that some soluble poisons, taken by the milk-giving animals, may be transmitted by the milk, and may set up poisonous symptoms in other animals partaking of such milk as food.

In relation to the effects of milk as conveying some forms of epidemic disorder, we have ourselves lately observed an isolated natural fact, which deserves a record notwithstanding its unity. At the Metropolitan Free Dispensary, there came before us, during the last month, a woman, who was barely convalescent from a very severe attack of erysipelas of the head and face. She had a child at her breast, which seemed well nourished and healthy. On inquiry, we found that six days after she had given birth to the child, the first symptoms of erysipelas set in. The disease manifested itself with extreme violence. Prior to the occurrence of this disorder, the milk secretion had been free; but with the first symptoms it decreased. The child, however, had no other nourishment. As recovery commenced, the milk returned as before, and the infant continued to subsist only on its natural food. It showed no indications whatever of disease during the course of these events, and at present remains well, the mother herself having made a perfect recovery. The milk of this woman, during the early stages of convalescence, was thin, pale blue in colour, and gave no cream after standing; microscopically it yielded no peculiar appearance.

In the *Annales d'Hygiène Publique*, vol. xxxv, there is an instance recorded, in which a child partook of the milk of a cow which had been bitten by a mad dog, and itself became rabid. The child suffered no injury.

It is not, however, to be presumed that escapes such as these are to be accepted as indicating that an infant can exist in health, on milk of an indifferent character. It is true that as yet we have no evidence to show that epidemic diseases can be conveyed through the breast-milk. But that many life disorders have their origin in this source, there is much circumstantial evidence in proof.

We have not the opportunity at this moment of entering at length into a wider range of new facts or theories on the question of diseased food and disease. But, as we have been appealed to many times in reference to various doubts and rumours, we can state the following conclusions:—

1. That at this time there is no specific typhus murrain in this country, analogous or identical with the murrain which has recently created such anxiety on the continent.

2. That the lung disorder, known now for some time in this kingdom, still exists, but not in a more intense form than at any other period.

3. That while there is no new specific disease in this country, there are in the unventilated, badly drained cattle sheds, with which the country abounds, all the favourable conditions for the continuance of diseases already present, and for the rapid dissemination of any new contagions or disorders which may spring up.

4. That if farmers would have less faith in cattle drinks, and more faith in pure air and water, and good food, they would save money, and the public would save health.

5. That as regards diseased animal food, and its application as food, there is no evidence to show that such food, when *well cooked*, is capable of conveying to the human subject any specific disorder; but this point is open to further investigation.

6. That the evidence is demonstrative that the consumption of some kinds of diseased food, in an imperfectly cooked state, leads to the generation of some specific forms of disease; such result being most marked in the development of tapeworms in the human subject, from the consumption of measly pork, and of some other varieties of flesh impregnated with entozoa.

7. That soluble poisons, taken by a milk-giving animal, may pass out of her system in part by the milk she secretes, and being taken by others in the milk thus secreted, may give rise to symptoms of poison. That milk, deficient in some of its constituent parts, or having some of these in excess, may interfere with nutrition, and create disorder of the alimentary functions, but that there are as yet but few proofs that the milk of a diseased cow used as food, possesses the power of producing any specific disease.

8. That owing to the chemical force of digestion, many organic agents, which would prove rapidly poisonous if sent direct into the blood, may be received through the stomach with impunity. But that the digestive process does not interfere with or prevent the development of the tapeworm, or of other forms of entozoa.

9. That the best protection for the masses against all possible mischiefs arising from bad meat, consists in their selecting their own provisions, in choosing the best, and in eating no animal food that is not well and cleanly cooked.

